# Acute Myocardial Infarction in a Young 26 Years Old Patient: A Rare Sequelae of Blunt Chest Trauma

**DOI:** 10.1002/ccr3.9606

**Published:** 2024-11-26

**Authors:** Muhammad Hanif, Muhammad Malik, Mostafa Vasigh, Abhigan Babu Shrestha, Debanik Chaudhuri

**Affiliations:** ^1^ Department of Internal Medicine, Division of Cardiovascular SUNY Upstate Medical University Syracuse New York USA; ^2^ M Abdur Rahim Medical College Dinajpur Bangladesh

**Keywords:** cardiovascular, coronary artery disease, myocardial infarction, trauma

## Abstract

Acute myocardial infarction (AMI) is an important subset of cardiovascular disease, and a medical emergency, where timely reperfusion is needed to reduce short‐term and long‐term complications from it. AMI following blunt chest trauma is a rare but serious complication of motor vehicle accidents and should be treated promptly. We are presenting a case of 26 years‐old male, who presented to ED after a motor vehicle accident, went into cardiac arrest, and was found ST elevated MI (STEMI) on electrocardiography. Subsequently, cardiac catheterization revealed ruptured plaque in the proximal left anterior descending artery, with thrombus extending to mid‐LAD, requiring a stent placement.

**Summary**

- Myocardial infarction after blunt chest trauma is a rare but serious complication and has rarely been reported after minor injuries such as mild sports injuries such as ball games.
- Every patient with blunt chest trauma should be investigated for cardiac injuries as early treatment of such injuries as myocardial infarction can prevent further hazardous effects and prevent long‐term permanent complications.

## 1 Introduction

Cardiovascular diseases (CVD) remain the leading cause of mortality in the United States and place a significant burden on the economy [1]. Myocardial infarction (MI) is one of the important subsets of CVD, which comprises a medical emergency and where every effort should be made to minimize the time needed to reperfuse the myocardium [2]. Acute MI following blunt chest trauma has been rarely reported in the literature and the first case of cardiovascular disease following blunt chest trauma was first described in 1764 by Akenside [3]. A multicenter study conducted by Alkhouli et al. reported that only 26% of all AMI reported following blunt chest trauma were STEMI, and the rest 76% were non‐STEMI [4]. Previous literature shows that most cases of AMI were reported immediately following motor vehicle accidents; however, rarely it has been reported after a week of blunt chest trauma [5]. Left anterior descending artery (LAD) has been reported as the most common injured vessel and intramural hematoma due to coronary contusion is the commonly reported mechanism of occlusion for causing AMI [6, 7]. Here, we are presenting a case of a 26‐year‐old male, who presented to the ED with complaints of neck pain and chest pain following a motor vehicle accident, went into cardiac arrest, reverted, and was found STEMI on EKG. Subsequently, cardiac catheterization revealed 90% ruptured plaque in the proximal left anterior descending artery (LAD), with thrombus extending to mid‐LAD, requiring a stent placement.

## 2 Case History/Examination

A 26‐year‐old male with a past medical history significant for marijuana use, presented to the emergency department with complaints of neck pain and chest pain following a motor vehicle accident. On examination, the patient's blood pressure was 125/93 mmHg, pulse of 110/min and regular, respiratory rate of 22/min, and was maintaining oxygen saturation of 98% at room air.

## 3 Methods

**FIGURE 1:**
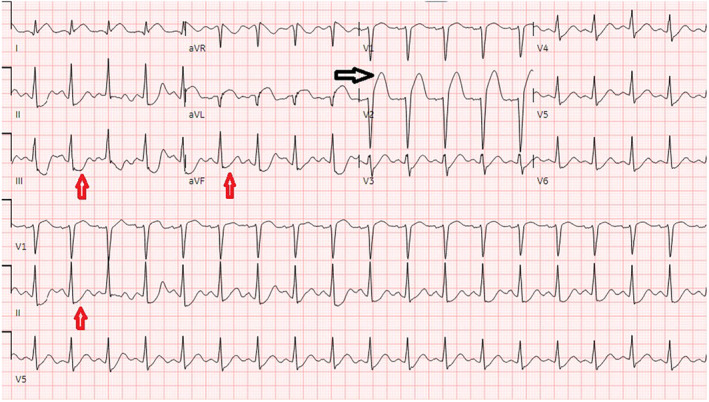
EKG showing ST elevation in anterior leads with reciprocal changes in inferior leads descending artery with thrombus extending into the mid‐LAD. Black arrow showing ST changes in anterior leads, while red arrows showing reciprocal changes in the inferior leads.

**FIGURE 2:**
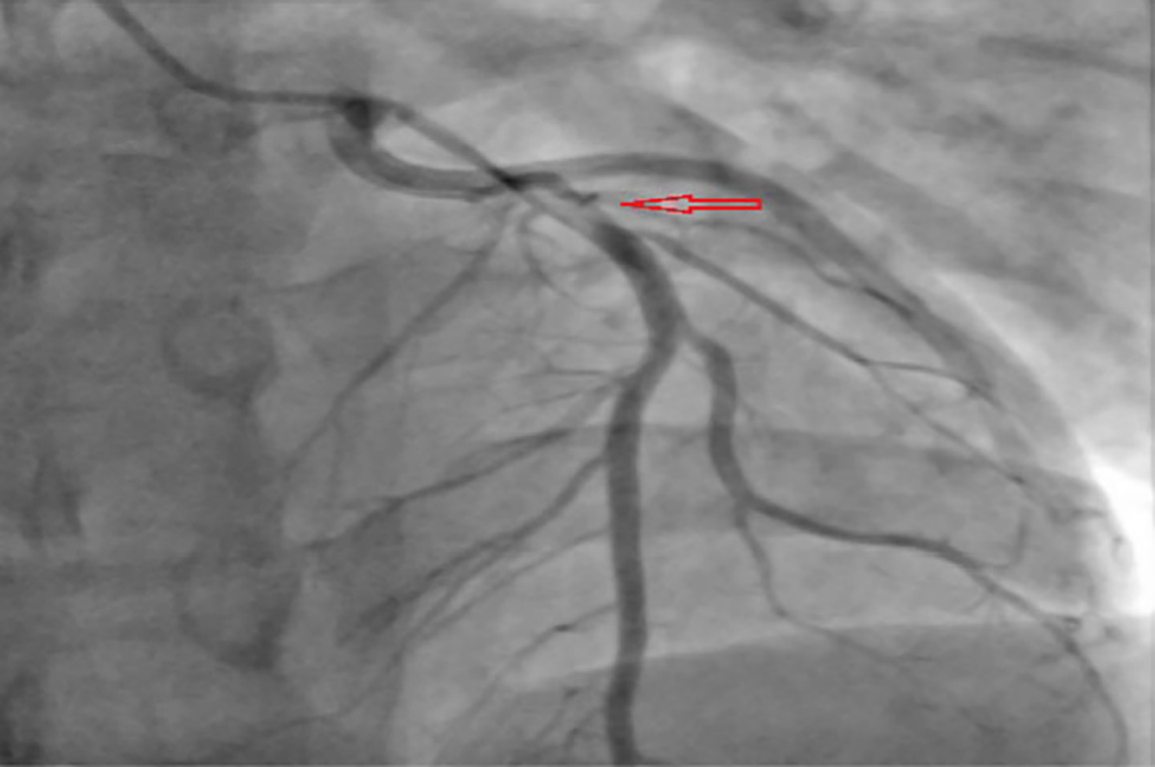
Coronary angiogram showing stenosis of the proximal left anterior (red arrow).

**FIGURE 3:**
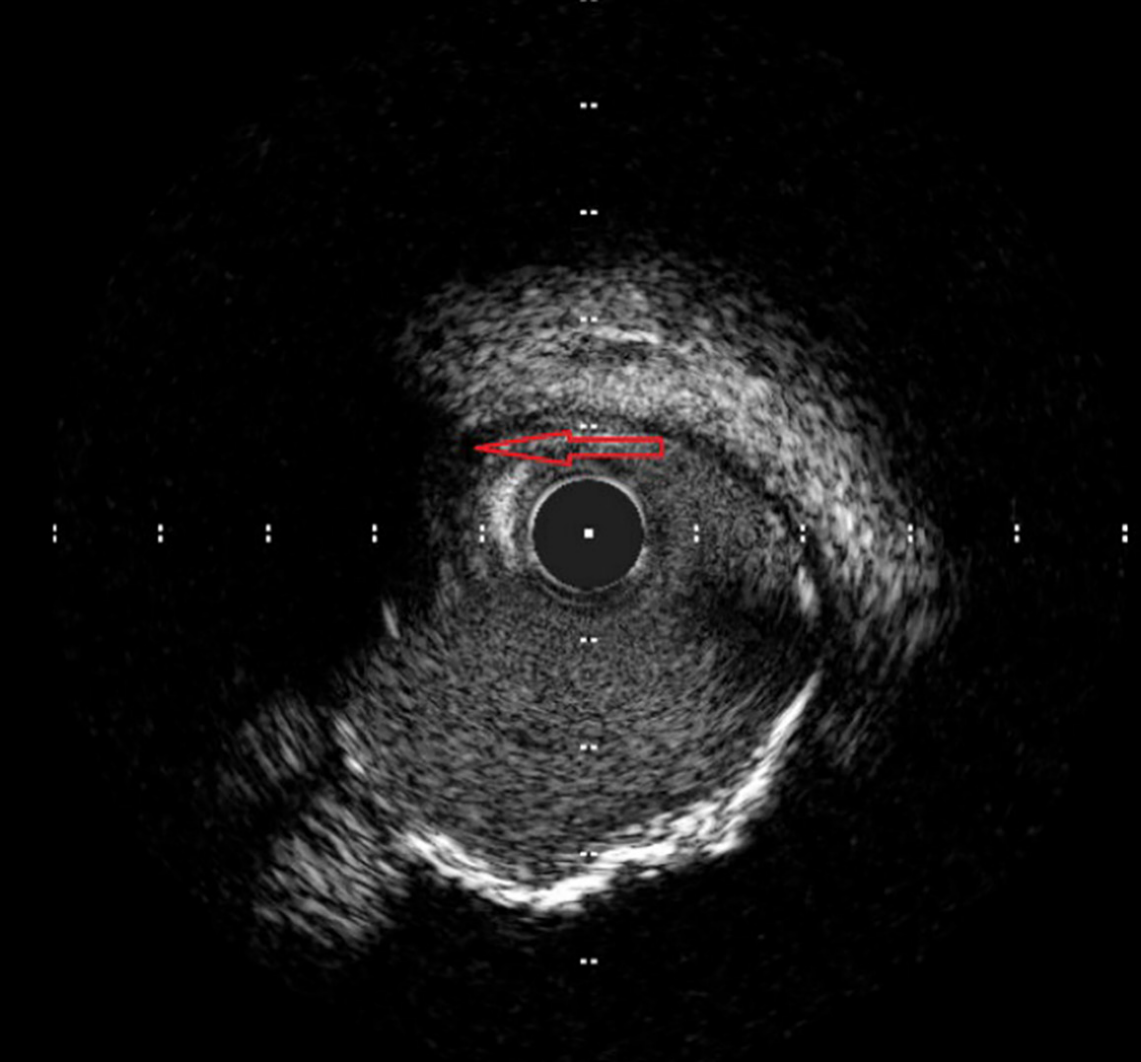
Intravascular ultrasound showing plaque with rupture (red arrow).

**FIGURE 4:**
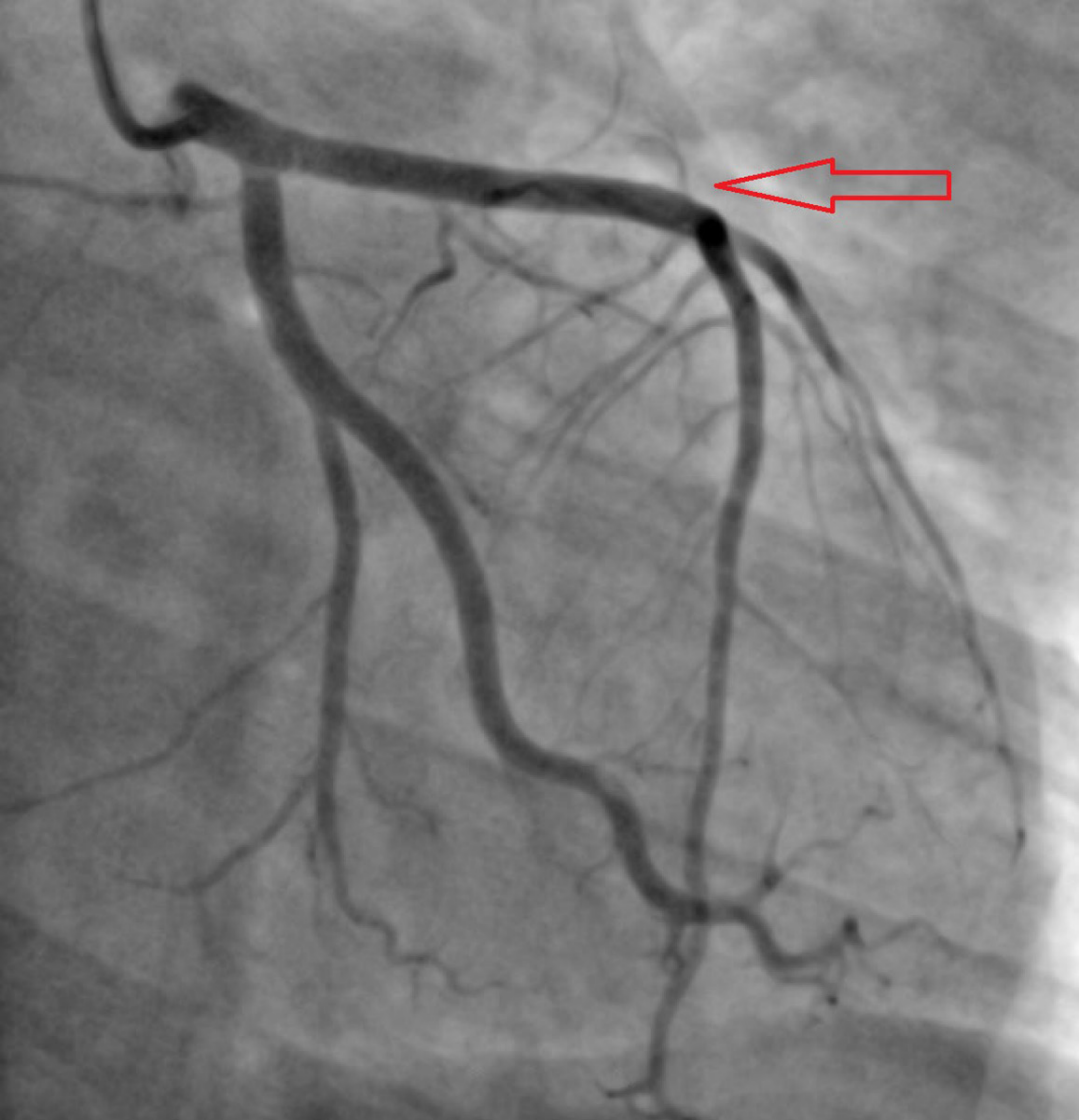
Post PCI angiogram showing the restoration of blood flow (red arrow).

Baseline investigations along with an x‐ray chest and an x‐ray pelvis were sent and reported negative for any fracture or acute cardiopulmonary disease. Baseline laboratory investigations were normal except for hypokalemia of 3.1 mmol/L (3–5 mmol/L), AST 167 U/L (8–48 U/L), ALT 128 U/L (8–55 U/L), serum lipase of 160 U/L (10–140 U/L), LDL of 96 mg/dL (< 100 mg/dL), and total cholesterol of 167 mg/dL (< 200 mg/dL). Contrast tomography (CT) head, cervical spine, thoracic, and lumbar without contrast was reported negative for any acute intracranial abnormality or acute traumatic injury involving the spine. CT thorax and abdomen pelvis were reported negative for any acute/traumatic abnormality. Blood toxicology reported positive for ethyl alcohol 0.17%, and high sensitivity troponin T levels were 28 ng/L (< 14 ng/L) on arrival. In the ED, the patient lost consciousness and was found in ventricular tachycardia, return of spontaneous circulation (ROSC) was achieved after two rounds of cardiopulmonary resuscitation and one shock of 120 J. Electrocardiogram reported sinus tachycardia and ST‐segment elevation concerned for acute anterior ST‐elevation myocardial infarction (STEMI) (Figure 1). Bedside transthoracic echocardiogram reported left ventricular ejection fraction 32%, and distal septum and anterior apex hypokinesia. STEMI code was activated, and the patient was transferred emergently to the cardiac catheterization laboratory. Cardiac catheterization revealed 90% stenosis of the proximal left anterior descending artery (LAD) with thrombus extending into the mid‐LAD with TIMI 1 flow distally (Figure 2). Intravascular ultrasound (IVUS) revealed a fatty plaque with acute rupture (Figure 3). The patient underwent successful IVUS‐guided percutaneous coronary intervention (PCI) to proximal‐mid LAD with a 3.0 × 38 mm Synergy drug‐eluting stent (DES) which was post‐dilated to 4.0 mm (Figure 4). Post‐procedure transthoracic echocardiogram reported LVEF of 56%, and distal to true apical anterior, mid to apical anteroseptal, and apical inferior wall hypokinesia. Overall, the apex was found severely hypokinetic to akinetic. The patient was started on dual antiplatelet therapy with aspirin and ticagrelor, high‐intensity statin, and beta‐blockers and was discharged home after 2 days and recommended follow‐up after 1 week.

## 4 Conclusion and Results

Myocardial infarction after blunt chest trauma is a rare but serious complication and has rarely been reported after minor injuries such as mild sports injuries such as ball games. Every patient with blunt chest trauma should be investigated for cardiac injuries as early treatment of such injuries as myocardial infarction can prevent further hazardous effects and prevent long‐term permanent complications. Emergency physicians and cardiologists should have low suspicion for cardiac injuries after blunt chest trauma, even while treating young patients without any cardiovascular risk factors.

## 5 Discussion

Myocardial infarction following blunt chest trauma is a rare but potentially serious complication and usually has been reported after severe blunt chest trauma like motor vehicle accidents, but rarely has been reported after mild sports injuries such as ball games in the literature [8, 9]. Various proposed mechanisms have been reported by which blunt chest trauma can damage the coronary arteries. A few of the possible mechanisms are; (a) shear stress applied to the coronary arteries causing intimal tears, which subsequently leads to intraluminal thrombosis, (b) coronary artery embolism, (c) vascular rupture due to blunt chest trauma, (d) vascular spasm due to adrenergic release after trauma, (e) fissuring of atherosclerotic plaque with dislodgment of plaque material [10].

According to the practice guidelines of the Eastern Association for the Surgery of Trauma, all patients with blunt cardiac injury (BCI) should undergo ECG on admission (level 1 evidence) [11]. Additionally, blunt cardiac injury can't be ruled out after normal EKG as most EKG changes after blunt trauma are transient, intermittent, and evolving and will require continuous telemetry monitoring despite normal EKG (level II evidence) [11]. In such patients, cardiac troponin‐I (highly sensitive and specific test) can play an important role in the differential diagnosis of cardiac injury [12]. Echocardiography is also important in patients with compromised hemodynamics to rule out mechanical complications such as cardiac tamponade, valve injury, or ventricle rupture [13]. Collins et al. revealed that normal EKG findings and negative cardiac troponin‐I findings 4–6 h have negative predictive value of 98%–100% post blunt trauma and almost completely rule out clinically significant blunt cardiac injury, and these patients can be safely discharged home [14].

The optimal medical management of AMI following blunt trauma is not clearly defined in the literature, probably because of lower incidents of such conditions, which makes it difficult for the researcher to conduct a large study trial. Most previous reports approached conservative therapeutic management, but thrombolytic treatment of coronary arteries has been successfully applied [15]. However, many trauma patients are not suitable candidates for thrombolytic due to the risk of hemorrhage from existing injuries. On the other hand, reperfusion in AMI following blunt chest trauma can also be achieved by performing percutaneous coronary intervention (PCI) like angioplasty or stenting [16]. Similarly, in our case patient was found to have STEMI after achieving ROSC from cardiac arrest, and successful timely PCI to proximal‐mid LAD with a drug‐eluting stent (DES) was performed. Timely reperfusion management is a key factor in treating AMI, as the benefits of reperfusion are the greatest if therapy is initiated early.

## Author Contributions

**Muhammad Hanif:** writing – original draft. **Muhammad Malik:** writing – original draft. **Mostafa Vasigh:** writing – original draft. **Abhigan Babu Shrestha:** writing – original draft, writing – review and editing. **Debanik Chaudhuri:** writing – original draft, writing – review and editing.

## Ethics Statement

The authors have nothing to report.

## Consent

Written informed consent was obtained from the patient for publication of this case report and accompanying images. A copy of the written consent is available for review by the Editor‐in‐Chief of this journal on request.

## Conflicts of Interest

The authors declare no conflicts of interest.

## Data Availability

Data available on request from the authors.

## References

[1] Global Burden of Cardiovascular Diseases Collaboration , G. A. Roth , C. O. Johnson , et al., “The Burden of Cardiovascular Diseases Among US States, 1990‐2016,” JAMA Cardiology 3, no. 5 (2018): 375–389, 10.1001/jamacardio.2018.0385. 29641820 PMC6145754

[2] S. Maxwell , “Emergency Management of Acute Myocardial Infarction,” British Journal of Clinical Pharmacology 48, no. 3 (1999): 284–298, 10.1046/j.1365-2125.1999.00998.x. 10510138 PMC2014343

[3] T. S. Marroush , A. V. Sharma , L. D. Saravolatz , R. Takla , and H. S. Rosman , “Myocardial Infarction Secondary to Blunt Chest Trauma,” American Journal of the Medical Sciences 355, no. 1 (2018): 88–93, 10.1016/j.amjms.2016.12.010. 29289269

[4] M. Alkhouli and F. Alqahtani , “Incidence and Outcomes of Acute Myocardial Infarction During Motor Vehicle Accident Related Hospitalizations,” American Journal of Cardiology 123, no. 5 (2019): 725–728, 10.1016/j.amjcard.2018.11.050. 30551839

[5] S. C. Vlay , D. S. Blumenthal , D. Shoback , K. Fehir , and B. H. Bulkley , “Delayed Acute Myocardial Infarction After Blunt Chest Trauma in a Young Woman,” American Heart Journal 100, no. 6 Pt 1 (1980): 907–916, 10.1016/0002-8703(80)90073-3. 7446393

[6] A. Boi , F. Sanna , A. Rossi , and B. Loi , “Acute Myocardial Infarction Secondary to Blunt Chest Trauma in Motorcycle Accident: A Rare Combination Where Percutaneous Coronary Intervention and Intravascular Imaging Optimization Are Needed,” Catheterization and Cardiovascular Interventions 92, no. 7 (2018): E456–E460, 10.1002/ccd.27725. 30208250

[7] M. M. James , M. Verhofste , C. Franklin , G. Beilman , and C. Goldman , “Dissection of the Left Main Coronary Artery After Blunt Thoracic Trauma: Case Report and Literature Review,” World Journal of Emergency Surgery: WJES 5 (2010): 21, 10.1186/1749-7922-5-21. 20649988 PMC2914738

[8] J. L. Marcum , D. C. Booth , and P. M. Sapin , “Acute Myocardial Infarction Caused by Blunt Chest Trauma: Successful Treatment by Direct Coronary Angioplasty,” American Heart Journal 132, no. 6 (1996): 1275–1277, 10.1016/s0002-8703(96)90475-5. 8969583

[9] J. E. Moore , “Acute Apical Myocardial Infarction After Blunt Chest Trauma Incurred During a Basketball Game,” Journal of the American Board of Family Practice 14, no. 3 (2001): 219–222. 11355055

[10] A. K. Sinha , R. K. Agrawal , A. Singh , et al., “Acute Myocardial Infarction due to Blunt Chest Trauma,” Indian Heart Journal 54, no. 6 (2002): 713–714. 12674188

[11] K. Clancy , C. Velopulos , J. W. Bilaniuk , et al., “Screening for Blunt Cardiac Injury: An Eastern Association for the Surgery of Trauma Practice Management Guideline,” Journal of Trauma and Acute Care Surgery 73, no. 5 Suppl 4 (2012): S301–S306, 10.1097/TA.0b013e318270193a. 23114485

[12] S. Agewall , E. Giannitsis , T. Jernberg , and H. Katus , “Troponin Elevation in Coronary vs. Non‐Coronary Disease,” European Heart Journal 32, no. 4 (2011): 404–411, 10.1093/eurheartj/ehq456. 21169615

[13] A. Renzulli , C. Wren , and C. J. Hilton , “Coronary Artery‐Left Ventricular Fistula and Multiple Ventricular Septal Defects due to Blunt Chest Trauma,” Thorax 44, no. 12 (1989): 1055–1056, 10.1136/thx.44.12.1055. 2617447 PMC1020888

[14] J. N. Collins , F. J. Cole , L. J. Weireter , J. L. Riblet , and L. D. Britt , “The Usefulness of Serum Troponin Levels in Evaluating Cardiac Injury,” American Surgeon 67, no. 9 (2001): 821–826. 11565757

[15] G. S. Ledley , S. Yazdanfar , O. Friedman , and M. N. Kotler , “Acute Thrombotic Coronary Occlusion Secondary to Chest Trauma Treated With Intracoronary Thrombolysis,” American Heart Journal 123, no. 2 (1992): 518–521, 10.1016/0002-8703(92)90670-q. 1736589

[16] R. R. Patil , D. Mane , and P. Jariwala , “Acute Myocardial Infarction Following Blunt Chest Trauma With Intracranial Bleed: A Rare Case Report,” Indian Heart Journal 65, no. 3 (2013): 311–314, 10.1016/j.ihj.2013.04.018. 23809387 PMC3861227

